# Dietary Cholesterol and Lipid Overload: Impact on Male Fertility

**DOI:** 10.1155/2019/4521786

**Published:** 2019-12-06

**Authors:** Fabrice Saez, Joël R. Drevet

**Affiliations:** Genetics, Reproduction, & Development (GReD) Laboratory, UMR CNRS 6293, INSERM U1103, Université Clermont Auvergne, 28 Place Henri Dunant, 63000 Clermont-Ferrand, France

## Abstract

Lipid metabolic disorders due to poor eating habits are on the rise in both developed and developing countries, with a negative impact of the “Western diet” on sperm count and quality. Dietary lipid imbalance can involve cholesterol, fatty acids, or both, under different pathophysiological conditions grouped under the term dyslipidemia. The general feature of dyslipidemia is the development of systemic oxidative stress, a well-known deleterious factor for the quality of male gametes and associated with infertility. Sperm are particularly rich in polyunsaturated fatty acids (PUFA), an important characteristic associated with normal sperm physiology and reproductive outcomes, but also targets of choice for oxidative thrust. This review focuses on the effects of dietary cholesterol or different fatty acid overload on sperm composition and function in both animals and humans. The links between oxidative stress induced by dyslipidemia and sperm dysfunction are then discussed, including possible preventive or therapeutic strategies to preserve gamete quality, longevity when stored in cryobanking, and male fertility.

## 1. Introduction

In mammals, the formation of sperm able to fertilize is a multistep process consisting of the production of gametes in the testicles and their subsequent maturation. These posttesticular events begin in the epididymis [1] and continue in the female genital tract, allowing a small number of selected male gametes to finally reach the oocyte for the ultimate purpose of fertilization [2, 3]. Many sperm molecular components and/or properties are modified by the posttesticular maturation events (reviewed in [4–6]). A remarkable feature of these modifications is that they are all dependent on indirect mechanisms, *i.e.*, interactions/exchanges between sperm cells and their environment. Indeed, sperm cells are “silent” cells due to a very high DNA/nucleus compaction that does not allow gene transcription. In addition, the final step of morphological differentiation of sperm cells during spermiogenesis leads to the exclusion of most if not all cytosolic organelles, thus limiting the cells' ability to support translation and protein synthesis. Ultimately, this deprives sperm of any adaptive response to stress. Sperm cells are therefore very sensitive to the composition of their environment. As pathological situations lead to changes in the composition of the milieu in which sperm evolve, they can induce sperm dysfunction and male sterility. The epididymal territory is important to consider in this regard, as this organ is highly irrigated by both blood and lymphatic vessels, thus exposing cells to systemic influences that are themselves dependent on environmental conditions [7]. The composition of the epididymal fluid, responsible for sperm maturation, is the result of selective filtration of blood components through the epididymal epithelium. The composition of the blood and the integrity of the epididymal epithelium [8] are therefore two important parameters that can affect male fertility. It has been demonstrated in rodents that the cell junctions maintaining the integrity of the epididymal epithelium were targets of toxins of different origins [8], possibly altering the normal posttesticular maturation process of sperm.

Dyslipidemia is a term referring to a group of different blood lipid imbalances such as the frequent pure hypercholesterolemia (30% of the cases), hypertriglyceridemia, combined hyperlipidemia, or isolated decrease of HDL-cholesterol [9]. The prevalence of dyslipidemia is about 45% among men in Western countries, as reported in Canada [10] and France [9]. Dyslipidemia is associated with different pathophysiological conditions such as metabolic syndrome, obesity, and cardiovascular diseases [11]. The negative impact of lipid metabolism disorders on fertility is now recognized, but there is a clear lack of knowledge about the underlying molecular mechanisms involved. This article proposes to examine the impact of dietary fat overload (cholesterol and other fats) on male fertility, with a particular focus on the links between dietary fat overload and oxidative stress.

## 2. Dietary Cholesterol and Male Infertility

### 2.1. Animal Models

The study of the relationships between dietary cholesterol intake and fertility is limited in men, so some information has been reported using animal models. Rabbits are interesting models because, unlike rodents, they are sensitive to a cholesterol-enriched diet and their lipid metabolism is closer to that of humans than that of mice. In this respect, it is one of the best models to study alterations in lipoprotein metabolism and atherosclerosis, a frequent cardiovascular consequence of dyslipidemia [12, 13]. The classic model consists in feeding rabbits a diet containing up to 2% cholesterol, which triggers a significant increase in plasma cholesterol levels and the lipoproteins *β*-VLDL (very low-density lipoprotein) derived from the liver, that are highly atherogenic. In the 1990s, two studies from the same group reported that rabbits fed a cholesterol-enriched diet (0.5%) showed (i) a significant decrease in the ability of sperm to undergo acrosomal reaction [14] and (ii) a significant increase in the concentration of filipin-sterol complexes in the plasma membrane of the acrosomal region, only in *cauda* epididymis sperm [15]. These two reports have shown that an overload of dietary cholesterol causes alteration of the acrosomal lipid domains when sperm pass through epididymal maturation. Plasma cholesterol levels were high when rabbits were fed a high cholesterol diet (HCD), but no differences were observed in seminal plasma cholesterol levels, sperm cholesterol levels, or even in the ratio of sperm cholesterol to phospholipids in *cauda* epididymis sperm. However, the molecular mechanisms behind these changes are still completely unknown.

A series of more recent publications using rabbits fed with HCD confirmed the previous results and further explored the phenotype of the spermatozoa. The animals were fed with a 0.05% cholesterol-enriched diet leading to dyslipidemia as the total cholesterol level in the serum was significantly increased. Under these conditions, the authors also found an increase in filipin-sterol complexes in the sperm acrosome region, associated with an increased percentage of morphologically abnormal sperm, reduced total motility, reduced ability to undergo normal capacitation (measured by tyrosine protein phosphorylation), and therefore, a reduced percentage of progesterone-induced acrosome-reacted spermatozoa [16]. In this study, the total sperm cholesterol content was increased when the rabbits were fed with the HCD. The authors showed in a complementary study that all the modified parameters described in rabbits fed with the HCD could be restored when they received a food supplement containing 7% olive oil [17]. The same group characterized HCD-fed rabbits as having abnormalities during the spermiogenesis process. These include a defective manchette, a temporary microtubular-based structure responsible for sperm elongation, which caused abnormal acrosome and nucleus development and inaccurate tail implantation [18]. These defects were shown to be due to an abnormal interaction between the manchette-acrosome complex and the membrane microdomains. Here, the authors demonstrated that a dietary intake of 7% olive oil combined with HCD could restore a normal phenotype [19]. Unfortunately, the authors did not propose any hypothesis on how olive oil dietary supplementation could act to restore a normal phenotype. The action of olive oil on sperm and testicles of hypercholesterolemic rabbits may be partly related to its ability to preserve the functional capacities of the membranes, probably due to the specific properties of the oleic acid contained in the oil [20]. In addition, olive oil also acts as an antioxidant due to its polyphenol content [21], which may be involved in limiting lipoperoxidative events as discussed below.

Data from other research groups, using rabbits as an animal model, provided additional information on the suspected causes of male fertility decline due to hypercholesterolemia, bringing forward a disruption of the integrity of the blood-testis barrier in 2% HCD-fed animals [22]. In a model obtained by feeding male rabbits a high-fat diet containing 0.5% cholesterol (and 4% peanut oil), severe dyslipidemia combining hypercholesterolemia, hypertriglyceridemia, and an increase in blood pressure were obtained, a situation very typical of metabolic syndrome [23]. In this study, sperm parameters were affected and a significant decrease in normal morphology, progressive motility, and total motility were observed in animals with the metabolic syndrome. The sperm cells were capacitation-deficient as measured by their ability to trigger the progesterone-induced acrosomal reaction. Finally, the cholesterol content of sperm cells had increased significantly in dyslipidemic animals.

*Overall, hypercholesterolemia in rabbits modifies sperm morphology and function in combination with changes in plasma membrane composition and dynamics. These alterations appear to be due to testicular and epididymal dysfunctions, with changes in the membrane lipids more likely due to epididymal maturation defects.*

Although rabbits are the gold standard for studying foodborne hypercholesterolemia, studies have also been conducted in rodents. A number of transgenic mice strains have been used to study the molecular regulation of intermediates in the cholesterol metabolism, whether or not associated with dietary intake. Mice were also used to study male infertility related to diet-induced obesity (for recent examples, read [24, 25]); however, this is out of the scope of this review. In our group, we developed a diet-induced posttesticular infertility model triggered by feeding 3-month-old male Liver-X-Receptor knockout mice (*Lxr α*; *β-/-*) for 4 weeks with a cholesterol-enriched diet (1.25%). This model underlined that the epididymis is very sensitive to circulating factors that can interfere with normal gamete maturation. Dietary cholesterol overload (well managed by wild-type mice) led in the transgenic mouse strain to sperm abnormalities similar to those described in hypercholesterolemic rabbits including morphological changes, decreased motility, and capacitation failure associated with abnormal sperm plasma membrane lipid composition resulting in dynamic dysfunctions [26, 27].

The impact of dietary cholesterol overload on male fertility has also been studied in rats. Male rats fed for 120 days with a hypercholesterolemic diet showed a significant reduction in secondary spermatocytes and spermatids associated with increased plasma LDL levels and the development of aortic atherosclerosis [28]. A diet enriched with cholesterol (400 mg/kg body weight) administered for 60 days to albino rats resulted in a significant reduction in sperm motility, epididymal epithelial cell height (both in *caput* and *cauda* epididymal regions), and seminal tubule and Leydig cell nucleus diameters associated with increased plasma cholesterol and triglycerides. A decreased number of implanted fetuses in females mated with hypercholesterolemic males was also reported [29]. The deleterious effects of oxidized LDL (oxLDL) on male reproductive function were demonstrated by comparing a high cholesterol diet (2%) to a high oxidized-cholesterol diet in male Wistar rats for 14 weeks. The most adverse impact was obtained with a diet high in oxidized cholesterol, which resulted in a very significant increase in circulating oxLDLs and a significant decrease in sperm count, motility, morphology, and viability compared to control and cholesterol-fed animals [30]. This study underlines the additional effect of dyslipidemia and oxidative stress, a point that will be further developed.

*An overload of dietary cholesterol has a very negative impact on the reproductive function of male mice and rats, in accordance with the data obtained on rabbits.*

### 2.2. Data in Humans and Clinical Management

The possible relationship between dietary cholesterol and semen characteristics is difficult to assess in humans. As infertility clinicians only see patients when dyslipidemia is established, it is therefore impossible to compare in the same individuals semen parameters in the predyslipidemia state. Nevertheless, data are available on pathophysiological conditions involving dyslipidemia, such as obesity and metabolic syndrome, but these clinical situations are not strictly specific to cholesterol overload. In these situations, it has however been clearly demonstrated that adverse effects on male fertility exist (examined by [31–33]). The data available in men regarding the relationship between plasma cholesterol levels and semen parameters are mainly from studies conducted to investigate the effect of cholesterol-lowering treatments. The effects of pravastatin (inhibitor of hydroxymethylglutaryl coenzyme A reductase, a rate-limiting enzyme in cholesterol biosynthesis) or cholestyramine (sequestering bile acids) were compared on semen parameters in 14 hypercholesterolemic men with high LDL plasma levels [34]. Both treatments surprisingly caused a decrease in sperm motility at 6 and 12 months, which was probably the result of a decrease in total cholesterol and LDL levels, not a specific side effect of one of the treatments. The decrease in LDL cholesterol can also affect the maturation of epididymal sperm, as sperm acquire motility during this process, a point that has not been studied by the authors. In another study, the same group found no evidence of clinically significant effects of simvastatin or pravastatin on multiple sperm quality parameters as well as on gonadal testosterone production or testosterone reserve [35]. Other studies reported that statins administered to hypercholesterolemic men had no significant effect on sperm parameters. For example, simvastatin has been shown to have no effect on sperm quality in terms of motility, concentration, viability, and morphology after 14 weeks of treatment [36]. This was also the case for the long-term effects of pravastatin evaluated in eight hypercholesterolemic patients [37]. These data are not consistent with the data obtained in rats since one study reported that concomitant administration of alpha-tocopherol and simvastatin to male hypercholesterolemic rats improved their reproductive efficacy and provided additional protection against fertility loss induced by hypercholesterolemia [38]. This may indicate that it is not possible to reproduce the nutritional conditions of animal models in humans, or that the number of subjects included in these studies is too small, or, else, that human testes and epididymides may be less sensitive than animal tissues to dietary cholesterol overload.

*Links between hypercholesterolemia and male sperm parameters are rare, so comparative and prospective studies with a large number of men are definitely needed. The evaluation should be expanded to include, in addition to the usual sperm parameters, capacitation tests, fertilization biomarkers, and in vitro fertilization (IVF) data. Only such studies could provide a better understanding of the effect of hypercholesterolemia on human male fertility.*

## 3. Lipid Overload and Oxidative Stress: Links, Consequences, and Clinical Management

Sperm cells are very sensitive to lipid peroxidation because of their high content of polyunsaturated fatty acids (PUFA), particularly docosahexaenoic acid (DHA, C-22:6*n*-3, a fatty acid containing six double bonds [39]). The modification of the fatty acid composition of sperm has been linked to sperm dysfunction and fertility disorders in many studies. For example, when human sperm were separated on a discontinuous Percoll gradient, the DHA content was significantly different in the sperm of all fractions [40], more mature sperm containing 2.5 times less DHA than slightly less mature cells. Even though the net sperm DHA content decreases in relation with sperm maturity, it is important to mention that DHA remains the major PUFA of sperm cells, and which content is systematically lower in sperm from infertile men [41]. This decrease in DHA is part of a decrease in global sperm PUFA levels associated with an increase in the *n*-6/*n*-3 ratio in sperm cells of oligo- and/or asthenozoospermic men, suggesting a link between fatty acid composition and infertility [41]. This causal relationship was established in a report showing that defective human sperm cells contained high levels of nonesterified unsaturated fatty acids that promoted the generation of reactive oxygen species (ROS) by their mitochondria, thus creating oxidative stress and a concomitant loss of functional competence [42]. Taking all these elements into account, it is therefore very likely that diet-induced lipid changes modify the composition and functions of sperm cells and promote oxidative damage. In support of this, a recent study described a negative association between body mass index (BMI), DHA, and palmitic acid levels in semen [43].

### 3.1. Dietary Lipids Modify Sperm Composition and Quality

#### 3.1.1. Animals

Dietary lipid supplementation has several objectives related to male fertility: (i) to improve male reproductive fertility and (ii) to promote sperm resistance to freeze-thaw procedures widely used in artificial insemination (AI). There is a fairly large amount of literature on these different topics, sometimes using *in vitro* supplementation rather than *in vivo* dietary intake. We will focus here on data on dietary intake and showing the consequences on the lipid composition of sperm.

Improving sperm quality has long been a subject of interest for the reproduction of agronomic species such as birds and cattle. A positive influence of dietary fatty acid supplementation on sperm composition was demonstrated in roosters with the observation that the proportion of *n*-3 fatty acids in sperm was increased (and inversely that of *n*-6 fatty acids was lower) when the males were fed salmon oil rather than corn oil. This observation was associated with higher fertility rates [44]. In consequence, the *n*-6/*n*-3 ratio appears to be an important parameter associated with rooster fertility, higher ratios being related to lower fertility. In addition, roosters fed a control diet or a diet containing corn oil, fish oil, or flaxseed oil showed different changes that were associated with the peculiar fatty acid composition of each lipid source (for example, flaxseed has the lowest *n*-6/*n*-3 ratio). However, it seems that other factor(s) can affect the sperm fatty acid composition as the lowest *n*-6/*n*-3 semen ratio was obtained with fish oil, but not with flaxseed [45]. Unfortunately, no fertility data was available in this study. Several reports deal with the impact of dietary fatty acid supplementation on sperm quality and reproductive performance in ageing roosters. Although various dietary supplements have been used, the consensual result of these studies is that *n*-3 polyunsaturated fatty acids have the strongest effect on the reproductive performance of ageing roosters with regard to AI [46, 47]. Overall, from these rooster studies, there is a clear relationship between dietary lipid supplementation and sperm fatty acid composition [46–48], with an increase in DHA systematically linked to improved reproductive performance [46, 47]. These data are consistent with the fact that the DHA content was found associated with optimal sperm maturation and ability to fertilize in human sperm, as already mentioned above.

Dietary supplementation is also widely used in large domestic mammals to improve their fertility. Bovine reproduction worldwide is mainly carried out using AI with frozen semen, which makes the fertilizing capacity of frozen-thawed sperm an economic challenge. Studies comparing the impact of different dietary fatty acid supplements on fresh and frozen-thawed bovine sperm are available. It appears that DHA-enriched oils or the use of nutraceuticals have systematically improved the quality of fresh semen after several weeks, at least regarding the classical motility parameter [49–51]. When the fatty acid composition of sperm was assessed, an increase in *n*-3 fatty acids was observed after DHA-rich oil supplementation [51, 52], with at least in one of these studies, a parallel increase in *n*-3 fatty acid plasma concentrations. In frozen-thawed sperm, analyzed from the same bulls, the results were not always consistent, with some studies showing no improvement in sperm quality [49], others showing a positive effect on motility parameters [51], or the percentage of sperm with intact acrosome [52]. A study reported that the lipid composition of sperm improved after the freeze-thaw procedure, with an increase in the proportion of *n*-3 PUFA in sperm from fish oil-supplemented bulls compared to control animals, in association with improved sperm quality assessed by viability, progressive motility, and morphology [50]. In all these studies, no data were available on the reproductive performance of the bulls. Another limitation of these studies is that they were conducted on relatively small numbers, since the number of bulls *per* group was generally between 6 and 15. With the exception of one study where the presence of intact acrosomes, membrane fluidity, and ROS generation were assessed by flow cytometry [52], only “classical” parameters were assessed such as motility (subjective or using CASA), viability, and sperm morphology giving no clues as to the real beneficial effect of these supplementation in terms of reproductive performance.

In laboratory models such as rodents, the impact of dietary lipids on sperm and/or reproductive performance was also examined. Male Wistar rats fed for three successive generations on a semipurified diet in which fat was provided *via* hydrogenated vegetable fat showed a reduced litter size and a decrease in the percentage of morphologically normal spermatozoa [53]. In rats too, the effects of the *n*-3/*n*-6 PUFA ratio on male reproductive performance were studied by feeding male Sprague-Dawley rats for 2 months with diets containing different *n*-3/*n*-6 ratios (ranging from 0.13, 0.40, 0.85, and 1.52 to 2.85) which ratios were obtained by mixing adequate amounts of linseed oil and soybean oil. It appears that sperm concentration, normal sperm head morphology, and motility were highest at a *n*-3/*n*-6 ratio of 1.52, in association with higher litter size and birth weight [54]. These data are consistent with the beneficial effects of *n*-3 fatty acid consumption on male reproductive capacity. They also show that an appropriate dietary ratio of *n*-3/*n*-6 PUFA is necessary for optimal male reproductive function. Unfortunately, this last study does not mention the *n*-3/*n*-6 ratio of sperm, which could have strengthened the link between dietary lipids and sperm composition, as suggested by other authors. In another study, Ferramosca *et al.* tested the hypothesis that supplementation of a high-fat diet with different sources of PUFA could improve rats' metabolic and reproductive parameters. By using either olive oil (as a source of monounsaturated fatty acids (MUFA)) or krill oil (for *n*-3 PUFA), they showed that olive oil partially neutralized the negative effects of a high-fat diet on sperm quality, increasing gamete mobility, reducing oxidative stress (lipoperoxidation), and slightly improving mitochondria function [55]. These data are in accordance with beneficial effects of olive oil demonstrated on spermatozoa from hypercholesterolemic rabbits, as described above [17, 19]. In mice, data on dietary lipid supplementation and its effect on sperm function are rare. Mice were mainly used to evaluate the effects of gene invalidation on fertility, sometimes in combination with dietary supplementation to restore a phenotype or to induce a pathophysiological situation. For example, in an acrylamide-induced oxidative stress model, the addition of corn oil or pork fat to the standard diet has been shown to have potentiating effects on the negative impacts observed on sperm cells and epididymal tissue. The addition of either of these two supplements resulted in a significant decrease in sperm concentration, mobility, and viability compared to mice solely exposed to acrylamide [56]. This decrease in sperm quality was accompanied by a significant increase in lipoperoxidation (as measured by the malondialdehyde (MDA) content of the sperm) and carbonylation of sperm proteins, as well as a significant decrease in primary antioxidant enzyme activities (superoxide dismutase (SOD) and glutathione peroxidase (GPx)). This study brings forward the potentiating effect of dietary lipids when an oxidative stress state exists. Finally, the importance of dietary fatty acids in male fertility was demonstrated by a study on mice invalidated for the enzyme delta-6 desaturase, the first rate-limiting enzyme in PUFA synthesis (*n*-3 and *n*-6 PUFA). Males of this strain showed infertility and spermatogenesis arrest at late spermiogenesis. Dietary supplementation with 0.2% DHA was sufficient to restore spermatogenesis and fertility [57]. Although the underlying molecular mechanisms explaining this phenotype and its rescue have not been described, DHA emerged as a central fatty acid in male fertility, as already mentioned earlier in this review.

*Dietary fatty acid intake has significant effects on gamete composition and male reproductive capacity. It is particularly important to maintain an *n*-3/*n*-6 ratio in an optimal range by modulating the *n*-3 intake.*

#### 3.1.2. Humans

The relationship between dietary fat and sperm quality has also been studied in men. In a study where diet was assessed using a dietary frequency questionnaire, the higher total fat intake was negatively correlated with total sperm count and concentration [58]. Dietary intake of saturated fat was the cause of this negative correlation; conversely, intake of *n*-3 fatty acids showed a positive correlation with normal sperm morphology. Dietary fats were weakly associated with sperm or seminal fatty acid levels, and there were only modest correlations between sperm, seminal fatty acid composition, and sperm quality. However, the authors reported that levels of saturated fatty acids in semen and seminal plasma were negatively correlated with sperm concentration and motility. This is in agreement with previous work showing higher concentrations of saturated fatty acids in the sperm of asthenozoospermic [41, 59] and oligozoospermic males compared to normozoospermic subjects [41]. Consistent with these data and animal data, Safarinejad *et al.* showed that *n*-3 PUFA blood and sperm levels were higher in fertile men than in infertile men with oligoasthenozoospermia. In these groups, the *n*-6/*n*-3 ratio of PUFA was significantly higher in the serum of infertile men [60]. Recently, the “FERTINUTS” study analyzed in healthy men aged 18-35 years the impact of a 60 g/day nut supplementation (30 g nut, 15 g almond, and 15 g hazelnut) on sperm DNA fragmentation; ROS production; chromosome X, Y, and 18 abnormalities; total DNA methylation; and microRNA content [61]. Walnuts contain about 50% fat, most of which is MUFA, with the exception of hazelnuts, where PUFA are the main fatty acids. After 14 weeks of nut supplementation, the authors reported several improvements over the control group in terms of sperm count, vitality, total motility, progressive motility, and morphology. Of all the other parameters evaluated, only the sperm DNA fragmentation was reduced in the “nuts” group. The authors concluded that “only a reduction in DNA fragmentation after nut consumption could explain these beneficial effects,” a point that seems questionable because it could be a collateral benefit of the absorption of antioxidant molecules such as vitamin E (as shown in supplementary table 7 of reference [61]). One of the limitations of this particular study is that dietary intake was only assessed by means of a questionnaire, and even if blood lipids were measured, there was no significant variation between the two groups for HDL, LDL, VLDL, total cholesterol, and triglycerides. In addition, fatty acids, and in particular *n*-3 and *n*-6, were not measured in blood or sperm, thus limiting the mechanistic relevance of nut supplementation to the fatty acid composition of sperm. However, eating nuts seems to be beneficial for human sperm because another study had previously reported improvements in sperm vitality, motility, and morphology when men took 75 g/d of walnuts for 12 weeks [62].

Clinical trials have also been initiated to investigate the potential effect of dietary PUFA on sperm quality in infertile patients. In a randomized, double-blind, placebo-controlled study analyzing the impact of 500 mg/day of DHA for 10 weeks, Martinez-Soto *et al*. showed no effect on traditional sperm parameters or lipid composition of the sperm membrane [63]. Interestingly, they however reported a significant decrease in the proportion of sperm with DNA damage in the DHA group as measured with the TUNEL assay using flow cytometry. They have no mechanistic explanation for this observation. Another study reported a positive effect of DHA supplementation on progressive sperm motility after 3 months with 0.5 or 1 g DHA/day [64]. A surprising result of this study was a significant increase in ROS sperm production in asthenozoospermic patients. This increase was however not accompanied by an increase in sperm lipoperoxidation, which led the authors to propose that DHA supplementation provokes a “positive” oxidative stress. Most recently, a review based on literature search tools examined the influence of DHA or EPA dietary supplementation, alone or with micronutrients, on sperm parameters in infertile and control men [65]. This analysis extracted three publications for a total of 147 infertile patients and 143 fertile controls, revealing a positive influence of *n*-3 fatty acids on total sperm motility and seminal plasma DHA concentration, without modification of sperm content.

*Overall, it appears that there is an influence of *n*-3 dietary fatty acids on sperm quality, which is not systematically associated with changes in the fatty acid profile of sperm. Mechanistic studies will be needed to understand the relative importance of testicular and posttesticular compartments in molecular changes associated with dietary fatty acid supplementation. Potential molecular regulators involved in the observed effects of *n*-3 PUFA supplementation are presently being examined [66].*

### 3.2. Oxidative Stress and Clinical Management

Spermatozoa are one of the body's richest cells in PUFA, which play a pivotal role in the regulation of their function, but this property also makes them very sensitive to oxidative stress. It is of the utmost importance that PUFA, typical of the plasma membrane of mammalian sperm and particularly sensitive to oxidation, are protected against lipoperoxidation. If this protection is not sufficient, the aldehydes resulting from the lipoperoxidation of the sperm plasma membrane will create a vicious circle of ROS amplification that will be very detrimental to the sperm structures and functions [67]. Although mature sperm cells are very susceptible to oxidative damage, paradoxically, sperm use ROS to complete their posttesticular maturation, particularly for optimal condensation of the sperm nucleus, a critical process that determines the level of integrity of paternal DNA [68]. In addition, sperm cells need ROS to complete major steps in the fertilization process, such as capacitation [69] and acrosomal reaction ([70] and reviewed by [71]). Oxidative stress is a characteristic of lipid metabolic disorders such as obesity and is related to subfertility (discussed in [72]). PUFA-based dietary fatty acid supplementation may be a good approach to overcoming sperm disorders, but it is pertinent to combine it with antioxidant supplementation to be more effective.

#### 3.2.1. Data on Animals

As mentioned above, dietary fatty acid supplementation can modify the PUFA composition of sperm cells, as shown in the work on roosters [44–46]. In some studies, the most important benefits observed on sperm parameters and fertility were obtained when dietary supplementation included vitamin E (Vit E) to protect sperm from lipoperoxidation [46, 47]. Combinations of canola oil, canola/fish oil or flaxseed oil, and 200 mg/kg Vit E were the most effective in improving AI results and sperm quality. The protective effects of Vit E supplementation have also been demonstrated in rats with improved sperm concentration and progressive motility [73]. Vitamin E supplementation has also been tested in bulls, but mainly in *in vitro* studies. It could improve sperm motility, membrane integrity, and oxidative stress after freezing and thawing of sperm when added as a complex with methyl-beta-cyclodextrin, a carrier in the cryopreservation medium [74]. Another study reported a protective effect on membrane lipids and better freezing of the bull's gametes when Vit E was added to the sperm extender [75]. Finally, a summary of the effects of *n*-3 PUFA dietary supplementation on rabbit reproductive parameters (male and female), with or without appropriate antioxidant protection (200 mg/kg vitamin E), has recently been published [76]. This study highlights the need for antioxidant supplementation in addition to dietary intake of *n*-3 PUFA to achieve beneficial reproductive effects in many species.

*Oxidative stress is harmful to male fertility in vivo and is a real problem for the preservation of sperm quality and DNA integrity in frozen samples used for AI. Although the benefits of different additives such as vitamin E have been reported, further studies are needed to improve the effectiveness of semen-freezing media.*

#### 3.2.2. Data on Humans

In men, structural and/or functional defects of sperm cells are due to multifactorial causes. Genetic, environmental, or lifestyle factors alone or in combination are at the roots of most infertility situations. Oxidative stress that alters sperm structures and functions is very often involved regardless of the cause of infertility. Oxidative damages to the sperm nucleus and in particular to paternal DNA have been detected in more than 60% of men visiting *in vitro* fertilization (IVF) centers, testifying of its prevalence [77]. Prevalence which is further increased in patients with idiopathic infertility, in whom this percentage rises to 80% [78]. Oxidative stress disrupts the integrity of sperm DNA but also damages the proteins and lipids in their plasma membranes, thus altering their fertility potential. To mitigate this damage, the use of antioxidant therapy has been suggested in the literature for many years [79–81] but there is still no consensus on its true clinical relevance due to the lack of adequate and rigorous clinical trials.

The most studied pathology combining lipid disorders, oxidative stress, and male infertility is obesity. In male mice, systemic oxidative stress induced by obesity was correlated with oxidative alterations in sperm DNA (as demonstrated by the TUNEL assay) and decreased fertility (measured *in vitro*) in males [82]. Paternal obesity or exposure to a high-fat diet also negatively affects the reproductive and metabolic health of the offspring, involving the alteration of sperm epigenetic marks such as DNA methylation and the sperm miRNA content. The results obtained in animal models have paved the way for the study of the effects of obesity on human sperm cells [83, 84]. Studies in men report that obesity causes a systemic inflammatory response that has negative consequences on sperm parameters and quality (reviewed in [85]). The inflammatory state induces the recruitment of white blood cells into male reproductive organs and/or seminal plasma, which leads to increased exposure of sperm to ROS and alterations in their genetic integrity. The pathophysiological situation of obesity is complex to analyze because it involves hormonal, inflammatory, and physical causes, all of which have a negative influence on male fertility. The underlying molecular dysfunctions are not well characterized, but more and more publications emphasize the importance of seriously addressing this pandemic, as the epigenetic legacy is now established [85–87].

Clinical management of male infertility should include good characterization of sperm abnormalities and special attention to paternal DNA damage to limit transgenerational effects. In addition, a better individual characterization would make it possible to propose appropriate therapies. In the case of obesity, it is known that a well-balanced diet associated with physical activity can improve sperm quality [88], but to date no data are available regarding the restoration of epigenetic markings. Limiting the oxidative stress encountered in obesity as in many other diseases seems to be a good strategy to improve sperm quality [77, 89]. However, to date, there is no consensus in infertility clinics, and various antioxidant treatments without scientific justification are given on a totally empirical basis. Each treatment must be adapted to the abnormalities of the individual's sperm, but in the absence of a detailed evaluation, clinicians are powerless to make a rational therapeutic choice. To this day, the evaluation of human fertility capacities is still based on WHO standards that only concern sperm concentration and morphology, which parameters are unanimously recognized as poorly predictive. Diet and supplementation have been shown to be effective in limiting oxidative damage to sperm, and the increased intake of different nutrients such as fruits and vegetables, selenium, zinc, *n*-3 fatty acids (mainly from fish and nuts), coenzyme Q10, and carnitines, alone or in combination have all been positively related to sperm quality [90, 91]. In this situation of lack of consensus and appropriate scientific and clinical data, antioxidant supplementation is going wild and could have deleterious effects for some patients. To date, there are more than 120 commercially available antioxidant formulations worldwide that claim to improve male fertility. None of them, except one [92], is supported by convincing scientific and clinical data. With regard to the particular profile of the sperm plasma membrane rich in PUFA, vitamin E supplementation should be the standard of excellence. However, some of these commercially available formulations do not even contain vitamin E, and for those that do, it is synthetic vitamin E, while nature has endowed mammalian cells with 8 natural isoforms of vitamin E, all of which are important to some extent. Knowing that there is only one cell membrane transporter for these vitamin E isoforms, it is easy to imagine what can happen when only one of the 8 is brought in excess. Preclinical data as well as rigorous, placebo-controlled, double-blind, multicenter clinical trials are absolutely necessary. We are confident that, in the case of established lipid disorders, individual testing for damage caused by the oxidation of sperm DNA will provide clinicians with the opportunity to propose intelligent and adapted therapeutic approaches that could avoid the use of assisted reproductive technologies. We also recommend that in situations of unexplained idiopathic infertility, especially in young men, the assessment of systemic lipid status should be part of the systematic male fertility examination.

*Diet can have a negative or positive effect on human fertility, as is the case for other major health problems (cardiovascular diseases, cancer, neurodegenerative pathologies, etc.). More research is needed to understand how diet can influence sperm parameters and fertility. A critical challenge will be to decipher the mechanistic actions of food supplements on male gametes in order to use them judiciously and in adapted pathophysiological situations. This is particularly important given the potential consequences that diet can have on the health of the offspring via epigenetic modifications.*

## 4. Conclusion

Dietary fats can influence the lipid composition of sperm and have harmful or beneficial consequences on male fertility (see Table 1 for an overview). This issue is of economic interest to the reproductive capacities of agricultural species such as birds and livestock. Dietary supplementation using a combination of *n*-3 PUFA (DHA) with antioxidant Vit E has shown the most beneficial effects to date. It is clear that further studies will be needed to understand the molecular mechanisms underlying *n*-3 PUFA supplementation. Dietary lipids are also of primary importance in human pathological situations, where their deleterious impact is associated with the concomitant onset of oxidative stress and sperm DNA damage. The increasing proportion of dyslipidemic men in the world raises a public health issue and a future challenge for infertility treatments because metabolic diseases are now considered as hereditary diseases *via* epigenetic mechanisms.

## Figures and Tables

**Table 1:** Summary of the main results reported in this review, showing the associated references.

Lipids involved	Main results and associated references			
	Animal models	Refs.	Humans	Refs.
*Cholesterol*	*Rabbits* fed a cholesterol-enriched diet (0.5%) present multiple sperm dysfunctions of epididymal origin due to membrane lipid microdomain alterations. High plasma cholesterol levels, but no changes in seminal plasma or sperm cholesterol levels.	[14, 15]	Studies on hypocholesterolemic compounds (statins, cholestyramine) gave heterogeneous results concerning sperm parameters.	[34–38]
	*Rabbits* fed with 0.05% cholesterol diet showed the same dysfunctions but with increased sperm cholesterol contents. A dietary supplementation with 7% olive oil restored normal characteristics.	[16, 17]		
	Sperm parameters were affected in *rabbits* with a dietary-triggered metabolic syndrome, deficiency in capacitation associated with an increase in sperm cholesterol levels.	[23]		
	Dietary cholesterol-induced dyslipidemia provokes posttesticular infertility in LXR-deficient *mice*.	[26, 27]		
	Cholesterol-enriched-diet altered sperm functions in *rats*.	[29]		

*Fatty acids*	Dietary supplementation with *n*-3 polyunsaturated fatty acids modifies the *n*-6/*n*-3 ratio and ameliorates sperm quality, fertility ratio, and reproductive performance of ageing *roosters* (artificial insemination). Increased amounts of DHA are associated with these improvements.	[44–47]	Nut consumption for several weeks improves sperm count, vitality, total motility, progressive motility, morphology, and sperm DNA fragmentation compared to the control group.	[32, 61]
	Dietary supplementation with DHA-enriched oils improves fresh and cryopreserved *bovine* sperm quality, in association with higher *n*-3 PUFA contents (for cryopreserved samples).	[49–52]	Dietary supplementation with 500 mg/day DHA for 10 weeks has no effect on sperm parameters or sperm membrane lipid composition.	[64]
	The *n*-3/*n*-6 ratio of dietary oils ameliorates reproductive outcomes of male *rats*.	[54]	A review based on literature search tools showed positive influence of dietary supplementation with *n*-3 fatty acids on total sperm motility and seminal plasma DHA concentration without modification of sperm content.	[65]
	Dietary supplementation with 0.2% DHA restores the infertility due to spermatogenesis arrest in D6-desaturase invalidated *mice*.	[57]		

*Links with oxidative stress*	The most important benefits observed on *rooster* sperm parameters and fertility were obtained when dietary supplementation included vitamin E to protect sperm from lipoperoxidation.	[46, 47]	Obesity causes a systemic inflammatory response that has negative consequences on sperm parameters and quality, associated with an increased exposure of sperm to ROS.	[85]
	Vitamin E supplementation is also efficient in *rats* and *in vitro* on *bull* spermatozoa.	[73, 74]	Diet (*n*-3 fatty acids from fish oil and nuts) and antioxidant supplementation have been shown to be effective in limiting oxidative damage to sperm.	[90, 91]
